# Understanding Antibiotic Use in Minya District, Egypt: Physician and Pharmacist Prescribing and the Factors Influencing Their Practices 

**DOI:** 10.3390/antibiotics3020233

**Published:** 2014-06-20

**Authors:** Kathleen L. Dooling, Amr Kandeel, Lauri A. Hicks, Waleed El-Shoubary, Khaled Fawzi, Yasser Kandeel, Ahmad Etman, Anna Leena Lohiniva, Maha Talaat

**Affiliations:** 1 Centers of Disease Control and Prevention, Atlanta, GA 30333, USA; E-Mails: kathleen.dooling@peelregion.ca (K.L.D.); auq3@cdc.gov (L.A.H.); 2 Ministry of Health and Population, Cairo 11516, Egypt; E-Mails: kandeelamr@yahoo.com (A.K.); kfawzy56@yahoo.com (K.F.); ykandeel@hotmail.com (Y.K.); ahmed.hetman@hotmail.com (A.E.); 3 U.S. Naval Medical Research Unit, N3, Cairo 11517, Egypt; E-Mails: waleed.el-shoubary.eg@med.navy.mil (W.E.-S.); anna.leena.lohiniva@hotmail.com (A.L.L.)

**Keywords:** antibiotics, antibiotic resistance, acute respiratory infection, cold, bronchitis, sinusitis, pneumonia, pharmacist, Egypt

## Abstract

Overuse of antibiotics has contributed to the emergence of antibiotic-resistant bacteria globally. In Egypt, patients can purchase antibiotics without a prescription, and we hypothesized frequent inappropriate antibiotic prescribing and dispensing. We interviewed physicians (n = 236) and pharmacists (n = 483) and conducted focus groups in Minya, Egypt, to assess attitudes and practices regarding antibiotic prescribing for outpatient acute respiratory infections (ARI). Antibiotics were reportedly prescribed most of the time or sometimes for colds by 150 (64%) physicians and 326 (81%) pharmacists. The most commonly prescribed antibiotics were β-lactams. Macrolides were the second most commonly prescribed for colds and sinusitis. The prescription of more than one antibiotic to treat pneumonia was reported by 85% of physicians. Most respondents thought antibiotic overuse contributes to resistance and reported “patient self-medication” as the biggest driver of overuse. Fifty physicians (21%) reported that they had prescribed antibiotics unnecessarily, citing patient over-the-counter access as the reason. Physicians <40 years of age and those who treat adults were more likely to prescribe antibiotics for colds. Overall, we found a high rate of unwarranted outpatient antibiotic prescribing and dispensing for ARIs. Patient access to OTC antibiotics contributes to over-prescribing. National guidelines for ARI treatment, provider education and national policy requiring a physician’s prescription for antibiotics may improve appropriate antibiotic use in Egypt.

## 1. Introduction

Inappropriate and excessive use of antibiotics and the resultant selective pressure on bacteria is an important mechanism contributing to the development of bacterial resistance [1,2,3]. In many middle-income countries, including Egypt, antibiotics are available and dispensed at pharmacies without a physician’s prescription [4]. A study in southern and eastern Mediterranean countries found that 30% of Egyptian participants had self-medicated with antibiotics during the previous year. This was more than 50% greater than other Mediterranean countries and almost six times the proportion reported from a similar European study [4,5]. In addition to self-medication, the rate of physician antibiotic prescription for common infections, such as mild acute respiratory infections (ARIs) that do not require antibiotics is extremely high in many countries [4,5,6].

Infections caused by antibiotic-resistant bacteria are associated with increased healthcare costs and increased morbidity and mortality [7]. Although antimicrobial-resistant infections and the associated costs have been more fully described in developed countries, resistance is a worldwide problem. For example, community spread of resistant *Streptococcus pneumoniae* has been described in southern and eastern Mediterranean countries, as well as in Asia, Mexico, Argentina, Brazil, Kenya and Uganda [8]. In Egypt, active surveillance for resistant infections in three Egyptian university hospitals revealed high rates of β-lactam and methicillin-resistant *Staphylococcus aureus* [9]. Antimicrobial resistance has the potential to result in greater health impact in low and middle income counties, where the infectious disease burden is high, but access to or ability to pay for expensive second-line antibiotics is limited.

ARIs account for the majority of visits for outpatient care, and they are the most common reason a patient receives an outpatient antibiotic prescription in the United States [10] or seeks antibiotics without a prescription in the Euro-Mediterranean Region [4,5]. However, there are no published studies that characterize antibiotic prescribing and dispensing practices by physicians and pharmacists in Egypt. We hypothesized that inappropriate antibiotic prescribing and dispensing is common in Egypt. The objective of this study was to assess the knowledge, attitudes and practices of physicians and pharmacists when treating outpatient ARIs in Minya District, Egypt.

## 2. Methods

### 2.1. Study Location

The study was conducted in Minya District (population 220,000), the capital of Minya Governorate, located 280 km south of Cairo in Upper Egypt. Minya was chosen as the study site because the health directorate had the necessary infrastructure to conduct surveys. The majority of the population lives in rural areas, where agriculture is the predominant economic activity. Minya District has a network of government healthcare facilities composed of tertiary-care hospitals, district hospitals, rural hospitals and primary healthcare units.

### 2.2. Study Population

A comprehensive list of physicians who provide care for outpatients with respiratory infections was provided by the Directorate of Health (DoH) in Minya District. The specialties included were: general practice, chest medicine, internal medicine, pediatrics and otolaryngology. After obtaining study approval from the U.S. Naval Medical Research Unit, No. 3 (NAMRU-3), and the Egyptian Ministry of Health and Population, all doctors in the five specialties (n = 254) were invited to participate in the survey via official letters from the DoH. During the days of the survey, a group of trained interviewers met the primary rural healthcare physicians in the DoH, whereas physicians working in private or government hospitals were visited at their workplaces for interviews.

A complete list of pharmacists employed at primary healthcare centers or hospitals was provided by the pharmacy department at the DoH. All 140 government pharmacists were informed of the study by official letter from the DoH. Interviews with pharmacists in primary health centers were conducted in the DoH, whereas hospital pharmacists were visited at their workplaces for interviews. During interview days at the healthcare facilities, all physician and pharmacists were again invited to participate via word of mouth, and respondents participated during their paid shift.

Accurate information was not available on the number of private pharmacies; therefore, a census was conducted to identify all private pharmacies in Minya District. The census was performed by pharmacists employed at the DoH in Minya by locating all storefronts in the community and asking owners if there were any others in the neighborhood. Overlap in the referral list and the census list indicated good coverage, but exact completeness was not known. Information was collected on 450 private pharmacies, including the location and number of employees. Interviews were conducted by seven trained study personnel. 

Physician consultations are free in the public system, and patients receive the antibiotics from the government pharmacy free of charge. However, wait times are long and consultations short in comparison to the private system, where patients pay for the consultation and for the medicine. Although antibiotics are available in private pharmacies without a physician’s prescription, patients who desire a medical evaluation and diagnosis may consult with a physician prior to purchasing antibiotics.

### 2.3. Survey

Following informed consent, we administered in-person questionnaires to physicians and pharmacists during April and May, 2011. Information gathered for both groups included age and sex, education, knowledge and attitudes regarding antibiotic resistance, as well as usual practices related to the treatment of ARIs (pharmacists were only asked about colds). Practitioners could respond if they always, sometimes, occasionally or never treat ARI syndromes with antibiotics. In addition, physicians were asked to describe their practice (medical specialty, government *vs.* private practice, patient volume) and answer multiple choice questions pertaining to how they would treat ARIs in the pediatric or adult population. Physicians responded to four clinical scenarios, including three syndromes that did not warrant antibiotic therapy (cold, bronchitis and sinusitis) and one syndrome that did (pneumonia).

We performed descriptive analyses and evaluated factors associated with a physician’s prescription of antibiotics for colds. We used chi-square to test for independence of dichotomous risk factors. We performed all analyses using SAS version 9.3 (SAS Institute Inc., Cary, NC, USA).

### 2.4. Qualitative Study

To understand the reasons for prescribing antibiotics for ARI, 20 in-depth interviews were conducted with individual physicians. Physicians were selected according to seniority levels and places of work; five recent graduates (less than two years of experience in clinical practice), five senior clinicians (more than 20 years of experience) and 10 primary rural health unit doctors were interviewed. An additional 20 interviews were conducted with pharmacists who work in both public and private pharmacies in Minya District and who agreed to be interviewed. An open-ended question guide with a set of probes was developed to systematically cover reasons for antibiotic prescription. Interviews were conducted by four social workers with experience in qualitative research. Audio-recorded interviews were translated from Egyptian Arabic to English and transcribed by a professional translator. The accuracy of the transcription was checked by a bi-lingual investigator by comparing the recordings to the transcripts. Thematic analysis was used to analyze the data [11]. The analytic process started with an overview of the interview transcripts, highlighting words, phrases and sentences in order to extract the initial set of reasons for prescribing or recommending antibiotics. The highlighted sections were jointly reviewed by a multi-disciplinary project team, including an epidemiologist, infection control practitioner and health communication specialist, who summarized and sorted the reasons for prescribing. The reasons were merged into larger categories that led to a set of themes.

## 3. Results and Discussion

### 3.1. Results

#### 3.1.1. Questionnaire

A total of 236 (93%) physicians and 483 (81%) pharmacists responded to their respective surveys (Table 1). While 138 (98%) pharmacists who work for the government participated, 345 (76%) private pharmacists participated. Of the pharmacist respondents, 176 (36%) were pharmacist assistants. Seventy-three (30%) physicians and 55 (11%) pharmacists worked in both government and private settings.

With respect to antibiotic treatment for colds, 150 (64%) physicians and 326 (81%) pharmacists reported prescribing antibiotics most of the time or sometimes (Table 2). Physicians prescribed antibiotics more frequently for bronchitis and sinusitis and most consistently for pneumonia. The most common antibiotic choice of physicians across all ARIs was a β-lactam. However, 20 (13%) physicians indicated that they prescribe macrolides most of the time for colds, and 49 (22%) use this class most of the time for sinusitis. The prescription of more than one antibiotic was reported as the usual practice for 85% of physicians when treating pneumonia. The duration of the treatment selected by most physicians ranged from >7 days for pneumonia to 3–5 days for the treatment of colds. Most pharmacists (n = 242, 60%) treated colds for 3–5 days.

**Table 1:** Characteristics of physicians and pharmacists, Minya District, Egypt, 2011.

Characteristic	Physicians		Pharmacists	
**No. of participants**	236		483	
**Female**	102 (43%)		211 (44%)	
**Median age (range)**	42 (23–71)		27 (18–68)	
**Employer**				
**Government only**	150		80	
**Private only**	14		348	
**Both government and private**	73		55	
**Median patient visits/day**				
**Government clinic**	55 (1–150)		50 (1–1000)	
**Private clinic**	5 (1–50)		50 (2–600)	
**Training/Education **	Internal	63 (27%)	Secondary	89 (18%)
	General	64 (27%)	University	
	Pediatrics	59 (25%)	Pharmacy	305 (63%)
	Other	50 (21%)	Other	85 (18%)

**Table 2:** Self-reported antibiotic prescribing practices for various acute respiratory infections (ARIs), physicians and pharmacists, Minya District, Egypt, 2011.

Prescribing practice	Colds		Bronchitis	Sinusitis	Pneumonia
	Pharmacists *	Physicians	Physicians		
Prescribing frequency ^†^					
Most times	44 (11%)	22 (9%)	156 (66%)	103 (44%)	209 (89%)
Sometimes	282 (70%)	128 (54%)	71 (30%)	117 (50%)	9 (4%)
Never	78 (19%)	84 (36%)	6 (3%)	12 (5%)	2 (1%)
Antibiotic choice ^‡^					
β-Lactam	327 (81%)	122 (81%)	183 (81%)	123 (56%)	167 (77%)
Tetracycline	4 (1%)	4 (3%)	4 (2%)	14 (6%)	1 (1%)
Quinolone	16 (4%)	3 (2%)	21 (9%)	34 (16%)	38 (17%)
Macrolide	12 (3%)	20 (13%)	19 (8%)	49 (22%)	10 (5%)
Other	45 (11%)	1 (1%)	0	0	1 (1%)
Days of treatment	3–5 (60%)	3–5 (60%)	5–7 (60%)	5–7 (55%)	>7 (48%)
No. who prescribe >1 Abx	NA	14 (9%)	85 (37%)	54 (25%)	184 (85%)

* Pharmacists were only asked about prescribing for colds; ^†^ 404 pharmacists and 234 to 232 physicians responded to these questions; ^‡^ includes only providers who responded affirmatively to prescribing frequency; NA = not asked; Abx = antibiotics.

While 229 (97%) physicians agreed that overuse of antibiotics can make bacteria more resistant to antibiotics, among pharmacists, 369 (78%) agreed and 84 (18%) were unsure. Pharmacists and physicians both reported that patient self-medication with antibiotics and the incorrect duration of treatment were the top contributing factors to resistance (Table 3). When asked if antibiotics are necessary when nasal discharge turns from yellow to green, 194 (82%) physicians and 307 (65%) pharmacists agreed or strongly agreed and 138 (30%) pharmacists were unsure. When asked if antibiotics were helpful in treating colds, 19 (8%) physicians and 164 (34%) pharmacists agreed or strongly agreed.

**Table 3:** Survey responses of physicians and pharmacists regarding factors that contribute to antibiotic resistance.

Factors contributing to resistance	Physicians (n = 236)		Pharmacists (n = 437)	
	N (%)	Rank	N (%)	Rank
Patient self-medication with antibiotics	202 (86)	1	310 (71)	1
Incorrect duration of treatment	156 (66)	2	304 (70)	2
Incorrect choice of antibiotics	130 (55)	4	228 (52)	3
Patient non-compliance	109 (46)	5	220 (50)	4
Inappropriate prescription by physicians	139 (59)	3	192 (44)	5
Inappropriate dispensing by pharmacist	102 (43)	6	117 (27)	6

Based on the recall of practice, physicians <40 years of age and those who treat adults were more likely to inappropriately prescribe antibiotics for colds than older physicians and pediatricians, respectively (Table 4). Having less than a university education was the only factor associated with recommending antibiotics for colds among pharmacists. Fifty (21%) physicians reported that they have prescribed antibiotics when not medically necessary, citing patient over-the-counter access to antibiotics as the most common reason for prescribing unnecessary antibiotics.

**Table 4:** Physician and pharmacist factors associated with self-reported antibiotic prescribing for colds.

Physician Characteristics	% Prescribe antibiotics	χ^2^ *p*	Pharmacist characteristics	% Prescribe antibiotics	χ^2^ *p*
Specialty			Training		
Pediatrics	46	<0.001	<post-secondary	92	0.01
Adult medicine	70		≥post-secondary	82	
Age			Age		
<40 years	72	0.03	<30 years	82	0.30
>40 years	58		>30 years	87	
Sex			Sex		
Female	59	0.14	Female	85	0.61
Male	68		Male	83	
No. patients visits/week			No. patients visits/day		
<100	64	0.98	<50	82	0.42
≥100	64		≥50	85	
Continuing education *			Continuing education		
Y	65	0.92	Y	84	0.67
N	64		N	83	

* Continuing professional education in the last year.

**Figure 1:**
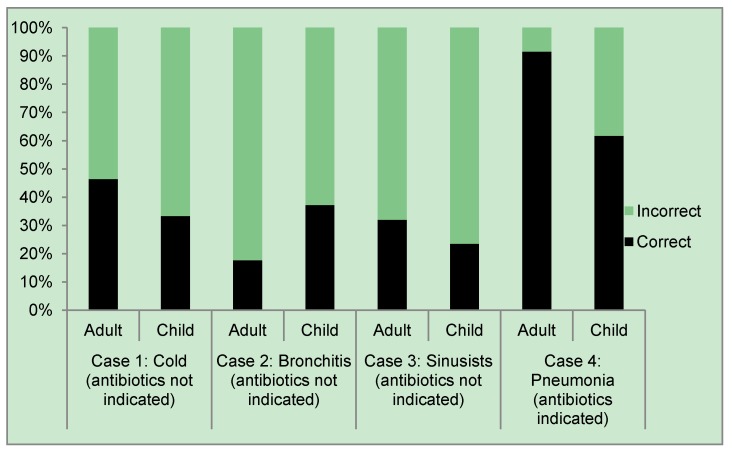
Physician treatment choices in response to acute respiratory infection (ARI) clinical scenarios.

When presented with clinical scenarios, most physicians answered that they would prescribe an antibiotic for colds, bronchitis and sinusitis—clinical scenarios for which antibiotic prescriptions were not indicated. A substantial minority of physicians (9% adult and 38% pediatric) withheld antibiotics for pneumonia—clinical scenarios for which antibiotics were indicated. The proportion of correct multiple choice responses to clinical scenarios ranged from 18% correct (no antibiotics necessary for acute bronchitis in an adult) to 92% correct (antibiotics necessary for pneumonia in an adult (Figure 1).

**Table:** 

Clinical case	Patient population	Correct	Incorrect
Case 1: Cold (antibiotics not indicated)	Adult	46.41	53.59
	Child	33.33	66.67
Case 2: Bronchitis (antibiotics not indicated)	Adult	17.65	82.35
	Child	37.25	62.75
Case 3: Sinusitis (antibiotics not indicated)	Adult	32.03	67.97
	Child	23.53	76.47
Case 4: Pneumonia (antibiotics indicated)	Adult	91.5	8.5
	Child	61.76	38.24

#### 3.1.2. Qualitative Study

##### 3.1.2.1. Physicians

In-depth interviews with physicians identified six important themes that influence the frequency and type of antibiotics prescribed: the unique unhygienic environment in Egypt, patient demand, lack of knowledge and guidelines, access and availability of antibiotics, as well as patient financial status.

The themes can be further categorized as factors pertaining to the physician or medical system *versus* factors pertaining to the patient. For example, physicians lacked knowledge on the role of antibiotics in treating viral infections. Only two physicians mentioned that antibiotics do not treat viral infections. Some physicians justified the choice not to treat mild ARIs with antibiotics by lack of illness severity rather than recognition of a likely viral etiology; many stated that guidelines for antibiotic use would be extremely helpful in clinical practice. Another system factor discussed was inequitable antibiotic availability by practice location. Physicians in rural health units often only had a few antibiotic agents available and, therefore, prescribed narrow spectrum antibiotics, if any. Hospital-affiliated physicians reported having slightly more antibiotic options than their rural counterparts. In the private clinics, however, physicians’ prescriptions would be filled by private pharmacies that stocked a wide array of antibiotics; therefore, new or multiple antibiotics may be prescribed. Many physicians reported that antibiotic resistance is a problem associated with the overuse of antibiotics. Physicians pointed out that their role in antibiotic consumption in Egypt is minor, as patients can buy antibiotics from any pharmacy without consultation. Another factor that influenced physician antibiotic prescribing was the belief that, in Egypt, unique unhygienic conditions put people at increased risk for infections. For example, one senior physician said that dust and wind in Egypt are different than any other country, and antibiotics were often required to cure resulting infections.

Physicians reported that patients frequently demand antibiotics and request certain types that had been effective in previous illnesses. Physicians usually comply with patient requests in order to maintain a good reputation. Finally, physicians noted that the selection of antibiotic agents depends on the financial status of the patient. Several physicians described that in the private sector, they prescribe expensive broad-spectrum antibiotics, because they believe patients who can afford them, prefer them.

##### 3.1.2.2. Pharmacists

Three main themes emerged that influence pharmacist dispensing of antibiotics: client demand, beliefs about the role of the pharmacist and attitudes about antibiotic resistance.

Most pharmacists agreed on the importance of gaining a reputation as a “clever pharmacist”—one who prescribes medications that work. Several pharmacists explained that to strengthen the client-pharmacist relationship, it is not sufficient to prescribe the same antibiotics; one must also offer new antibiotics. With respect to provider factors in dispensing antibiotics, pharmacists believed that it was both difficult and unnecessary to try to convince clients to choose other types of antibiotics. The pharmacists’ primary objective was to make financial profit and gain loyal clients. Finally, most pharmacists explained that resistance was due to the improper use of antibiotics among community members. Some of them mentioned that resistance is annoying, because it required them to continuously sell new medications. Antibiotic resistance was not considered to be a public health threat.

#### 3.2. Discussion

The majority of physicians and pharmacists report antibiotic prescribing for the common cold, a viral ARI for which antibiotic treatment is never indicated. Although a higher proportion of physicians than pharmacists report that they never prescribe antibiotics for colds, the antibiotic choice and duration of treatment is very similar for the two provider groups. Our survey results were reinforced by respondents’ articulation of the drivers of prescribing during in-depth interviews, such as physicians’ desire to build a good reputation and pharmacists’ desire to be regarded as being clever. Most respondents reported that they treated colds for a shorter duration (3–5 days) than other ARIs, indicating that they consider these infections to be less severe.

Younger physicians and those who saw adult patients were more likely than their counterparts to inappropriately prescribe antibiotics for colds. Younger physicians may be seeking to build their practice by satisfying patient requests for antibiotics. Many physicians working in governmental facilities also have private clinics, and physicians noted that a good reputation is essential to build a private practice. It is unclear why physicians caring for adults were more likely to prescribe inappropriately. The World Health Organization’s Integrated Management of Childhood Illness (IMCI) guidelines may help pediatricians decide when antibiotics are necessary [12,13]. As mentioned during the in-depth interviews, national guidelines for antibiotic use would be extremely helpful for all physicians. Specifically, education regarding the appropriate prescription of antibiotics should target medical students and those in early practice. However, given the system and financial pressures to build a private practice, it is unlikely that education alone will eliminate the over-prescribing of antibiotics.

In addition to overprescribing for colds, there is also likely to be some under-prescribing for pneumonia, based on physician responses to the clinical scenario multiple choice questions. Although only 5% of physicians reported that they sometimes or never prescribe antibiotics for pneumonia, 9% and 38% withheld antibiotics in adult and pediatric pneumonia scenarios for which antibiotics were indicated. Similarly, in a study characterizing Filipino physician prescribing, researchers found evidence of both over-prescribing for colds and under-prescribing of antibiotics for pneumonia [14]. The consequences of under-prescribing for pneumonia require careful consideration, as pneumonia accounts for almost one in five deaths in children under the age of five years and 1.6 million deaths in adults older than 65 years worldwide each year [15,16].

Survey and interview data revealed that most healthcare providers recognize that overuse of antibiotics can lead to bacterial antibiotic resistance. Unfortunately, because of the lack of access to microbiological testing, physicians may under-appreciate the prevalence of resistant bacterial infections. The majority of physicians and pharmacists reported that patient self-medication is the biggest driver of antibiotic resistance. One can derive from this shared response the provider sentiment of insufficient agency. Our qualitative research confirmed that providers perceived that antibiotic use is primarily in the hands of patients, as they act independently of physicians’ and pharmacists’ recommendations. By comparison, a recent qualitative study of factors influencing antibiotic prescribing among outpatient physicians in Germany found that, in addition to patient demand and self-medication, physicians also frequently recognized their responsibility to prevent antibiotic resistance in their own practice [17].

Our study had a number of limitations. The in-person surveys and in-depth interviews relied on self-reporting, which may have resulted in social desirability bias. However, we observed fair agreement between the physician answers to the clinical vignettes and physician self-reporting of prescribing for colds. Providers were asked to describe their antibiotic prescribing frequency, and categories of “some of the time” and “occasionally” were not specifically defined, which may have led to misclassification or misinterpretation. There are no clinical guidelines for the treatment of ARIs in Egypt; therefore, correct responses to clinical vignettes were based on practice standards from the United States and IMCI. Due to the high response rates, we believe our results are generalizable to Minya, but may not be generalizable to all of Egypt.

## 4. Conclusions

In conclusion, unwarranted antibiotic prescribing and dispensing for outpatients with ARIs is common among both physicians and pharmacists in Minya District, Egypt. Provider education regarding the indications for antibiotic prescribing and the consequences of overuse, as well as underuse is needed. The development and dissemination of national guidelines for the treatment of ARIs would be a useful first step to assist clinicians in making evidence-based choices regarding antibiotic therapy. Consideration should be given to a national policy that would require a patient to receive a physician’s prescription to obtain antibiotics. These study results are being used to develop educational interventions to improve antibiotic use.
